# Specific DNA binding of archaeal histones HMfA and HMfB

**DOI:** 10.3389/fmicb.2023.1166608

**Published:** 2023-04-18

**Authors:** Amanda M. Erkelens, Bram Henneman, Ramon A. van der Valk, Nancy C. S. Kirolos, Remus T. Dame

**Affiliations:** ^1^Leiden Institute of Chemistry, Leiden University, Leiden, Netherlands; ^2^Centre for Microbial Cell Biology, Leiden University, Leiden, Netherlands

**Keywords:** HMfA, HMfB, hypernucleosome, high-affinity DNA sequence, archaeal chromatin, archaeal nucleosome, genome organization

## Abstract

In archaea, histones play a role in genome compaction and are involved in transcription regulation. Whereas archaeal histones bind DNA without sequence specificity, they bind preferentially to DNA containing repeats of alternating A/T and G/C motifs. These motifs are also present on the artificial sequence “Clone20,” a high-affinity model sequence for binding of the histones from *Methanothermus fervidus*. Here, we investigate the binding of HMfA and HMfB to Clone20 DNA. We show that specific binding at low protein concentrations (<30 nM) yields a modest level of DNA compaction, attributed to tetrameric nucleosome formation, whereas nonspecific binding strongly compacts DNA. We also demonstrate that histones impaired in hypernucleosome formation are still able to recognize the Clone20 sequence. Histone tetramers indeed exhibit a higher binding affinity for Clone20 than nonspecific DNA. Our results indicate that a high-affinity DNA sequence does not act as a nucleation site, but is bound by a tetramer which we propose is geometrically different from the hypernucleosome. Such a mode of histone binding might permit sequence-driven modulation of hypernucleosome size. These findings might be extrapolated to histone variants that do not form hypernucleosomes. Versatile binding modes of histones could provide a platform for functional interplay between genome compaction and transcription.

## 1. Introduction

Every organism needs to compact its genome dynamically. Eukaryotes express histone proteins that form a defined octameric core with ~147 bp DNA wrapped around it, called the nucleosome (Luger et al., 1997). Archaea express histone homologs, which are involved in genome compaction and transcription regulation (Sandman and Reeve, 2005; Wilkinson et al., 2010). Together with other architectural proteins, such as Alba and MC1, archaeal histones have been hypothesized to function as transcription regulators (Henneman and Dame, 2015; Peeters et al., 2015). Expression of model histones HMfA and HMfB from *Methanothermus fervidus* in *Escherichia coli* resulted in a mild generic repressive effect on transcription (Rojec et al., 2019). Also, in their native environment, the histones of *Thermococcus kodakarensis* were shown to repress transcription, which was dependent on their multimerization state (Sanders et al., 2021). Archaeal histones are dimers in solution, although micrococcal nuclease (MNase) digestion studies in *M. fervidus, Haloferax volcanii* and *Methanobacterium thermoautotrophicum* point to a tetramer as the smallest relevant unit on DNA, showing protection of ~60 bp (Pereira et al., 1997; Ammar et al., 2012) Similar studies in *T. kodakarensis*, however, show protection of DNA increases with a length of ~30 bp steps up to 450 bp, suggesting multimerization adding dimers (Maruyama et al., 2013).

This multimer of archaeal histone dimers, called the hypernucleosome, is a rod-like structure with DNA wrapped around it (Mattiroli et al., 2017; Henneman et al., 2021). The formation of a hypernucleosome coats and compacts the DNA and could potentially play an important role in transcription regulation. Assembly of histone dimers into a hypernucleosome is dependent on stacking interactions between a dimer and its second and third neighbor (Henneman et al., 2018b, 2021). Most histones throughout the archaeal domain are predicted to be able to form hypernucleosomes, but some archaea encode histones that lack some or all stacking interactions (Henneman et al., 2018b). As archaea encode up to 11 histone variants within a single genome, many different combinations of dimers, tetramers and multimers are possible. Depending on different expression levels during the growth cycle and environmental cues, heteromerization could therefore play an essential role in modulating (hyper)nucleosome size and structure, potentially affecting transcription (Sandman et al., 1994; Musgrave et al., 2000; Marc et al., 2002). Histone variants lacking stacking interactions could act as ‘capstones’ and limit the size of hypernucleosome (Stevens et al., 2020).

Archaeal histones, like their eukaryotic counterparts, bind DNA without sequence specificity, but with a preference for more GC-rich sequences (Ammar et al., 2012; Warnecke et al., 2013). Transcription start sites (TSSs) are often AT-rich and are depleted from histones, both in archaea and eukaryotes (Segal and Widom, 2009; Nalabothula et al., 2013). HMfB was reported to preferentially bind GC-rich sequences with alternating GC and AT motifs (Pereira and Reeve, 1999; Bailey et al., 2000). Such a sequence motif also positions histone tetramers on genomic DNA in *H. volcanii* (Ammar et al., 2012; Warnecke et al., 2013). Using systematic evolution of ligands by exponential enrichment (SELEX), sequences with high affinity for HMfB were identified. One of the resulting sequences, “Clone20,” consists of alternating A/T-and G/C-rich regions (see Materials and Methods) and has a high binding affinity for HMfA and HMfB tetramers (Bailey et al., 2002). However, it is unclear whether such a high-affinity site functions as a nucleation site for hypernucleosome formation.

Here we show that HMfA and HMfB modestly compact Clone20 DNA by forming a tetrameric complex before hypernucleosome formation and that histone derivatives with impaired stacking interactions are still able to recognize the Clone20 sequence. High-affinity sites are likely bound by a geometrically different, more closed, tetramer, which is incompatible with hypernucleosome formation. This might indicate a previously unknown ability of histone variants that lack stacking interactions as tetrameric roadblocks halting hypernucleosome progression.

## 2. Materials and methods

### 2.1. Protein expression and purification

HMfA and HMfB were kindly provided by John Reeve and Kathleen Sandman. HMfA_K31A E35A_ and HMfB_D14A K30A E34A_ were purified as previously described (Henneman et al., 2021). Identity of the proteins was confirmed with mass spectrometry. Plasmids pRD323 (HMfA_K31A E35A_) and pRD324 (HMfA_D14A K30A E34A_) for expression of mutated HMfA and HMfB derivatives were deposited at Addgene with ID 198044 and 198045, respectively.

### 2.2. DNA substrate preparation

For the Tethered Particle Motion (TPM) DNA substrate, the Clone20 sequence (GCACAGTTGAGCGATCAAAAACGCCGTAGAACGCTTTAATTGATAATCAAAGGCCGCAGA, (Bailey et al., 2000)) was cloned into pBR322 using restriction digestion with EcoRI and HindIII (Thermo Scientific) resulting in plasmid pRD120. The same approach was used to create pRD123 containing Clone20R. Gibson assembly was used to create pRD196 containing Clone20L (Gibson et al., 2009). We used PCR to generate and amplify a 685 bp linear substrate containing the cloned sequence, using digoxygenin-and biotin-labeled oligonucleotides and DreamTaq DNA polymerase (Thermo Scientific) (van der Valk et al., 2017) or Phusion® High-fidelity DNA polymerase (Thermo Scientific). The products were purified with the GenElute PCR Clean-up kit (Sigma Aldrich). The nonspecific DNA substrate was prepared as previously described (Henneman et al., 2018a).

For microscale thermophoresis, 78 bp complementary oligonucleotides were designed using the Nonspecific and Clone20 sequence (Supplementary Table S1). The top strand was labeled with Cy5 and the complementary oligonucleotides were mixed 1:1 to a final concentration of 40 μM. Subsequently, they were heated to 95°C and slowly cooled to room temperature to anneal the strands.

### 2.3. Tethered particle motion

The tethered particle motion experiments, data analysis and representation of results were performed as previously described (Henneman et al., 2018a). To select single-tethered beads, we used a standard deviation cut-off of 8% and an anisotropic ratio cut-off of 1.3. As measurement buffer 50 mM Tris–HCl pH 7, 75 mM KCl was used.

The end-to-end distance was calculated by selecting the 25 beads closest to the fitted RMS at the respective protein concentration. Next, the 2.5% most distant positions of each bead were collected. The end-to-end distance was calculated for each point using triangular calculations and the diameter of the beads (0.44 μm). Next, the data was represented as histograms and fitted with a skewed Gaussian fit. The difference between the two populations was obtained by taking a pairwise distance distribution and fitting the resulting histogram with a Gaussian distribution.

### 2.4. Microscale thermophoresis

The DNA substrates described above with a concentration of 40 nM were diluted 1:1 with the HMf proteins. The final experimental buffer consisted of 50 mM Tris–HCl pH 8, 75 mM KCl. In MST experiments with HMfB_D14A K30A E34A_, 0.2% Tween20 was added for optimal solubility of the protein. The samples were incubated for 5 min at room temperature and transferred to MST capillaries (Monolith NT.115 Premium Capillaries, NanoTemper, Germany). The measurement was done at 40% LED power and medium MST power using the NanoTemper Monolith NT.115. Total measurement time was 40 s, with 5 s laser off, 30 s laser on and 5 s laser off. F_norm_ values were evaluated after 20 s of laser on. ΔF_norm_ values were calculated by subtracting F_norm_ of DNA only. Occupancy values were calculated and fitted with a Hill binding model.

### 2.5. Size exclusion chromatography with multi-angle light scattering

The molecular weight of HMf complexes in solution was measured using a SEC-MALS system comprising a miniDAWN® TREOS®, NanoStar DLS, Optilab differential refractometer (Wyatt technology) and 1,260 Infinity II multiple wavelength absorbance detector (Agilent). The samples containing at least 1 mg/ml HMfA or HMfB were run on a Superdex75 10/300 Increase GL column (Cytiva) with phosphate-buffered saline (12 mM NaPO4 pH 7.4, 137 mM NaCl) as running buffer. The ASTRA 8 software package was used to select the peaks and report the molecular weight.

## 3. Results

### 3.1. HMfA and HMfB bind as tetramers to the Clone20 sequence before hypernucleosome formation

**Figure 1:**
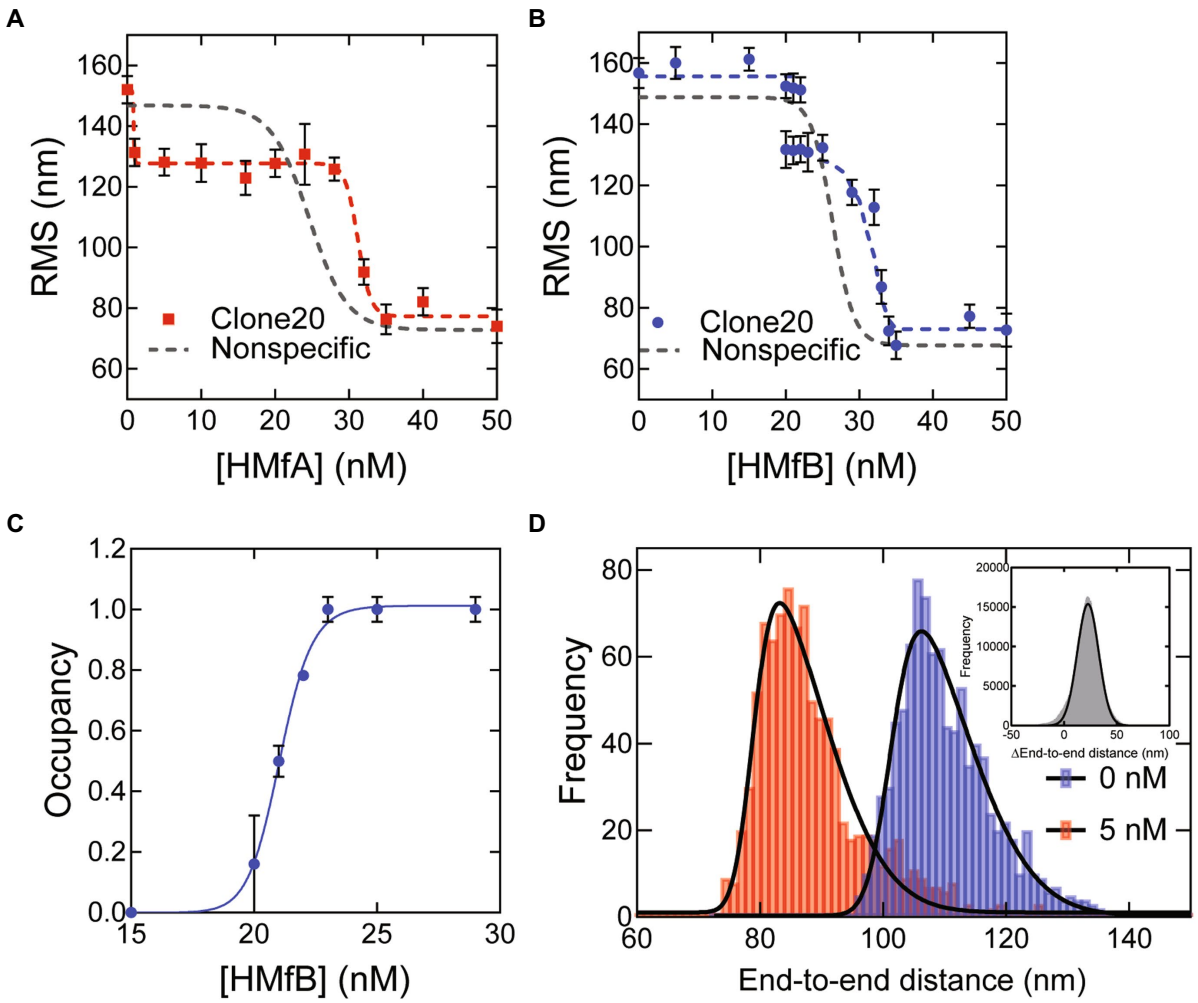
HMfA and HMfB bind as tetramers to the Clone20 site preceding hypernucleosome formation. **(A)** Root mean square displacement (RMS) values of Nonspecific and Clone20 DNA tethers incubated with HMfA and **(B)** with HMfB measured by TPM in 50 mM Tris–HCl pH 7, 75 mM KCl. Histograms were fitted with a Gaussian function and the mean values are represented by red and blue dots, respectively. Data for Nonspecific DNA was reproduced from Henneman et al. (2021) and depicted as a line to guide the eye. Error bars represent the propagated standard deviation of at least two replicates **(C)** Binding curve for specific binding of HMfB to the Clone20 substrate. The data points were fitted using the Hill binding model. Error bars represent the standard deviation of the replicates and propagated error for data points at saturation. **(D)** Calculated end-to-end distances for unbound Clone20 DNA and with 5 nM HMfA. Histograms were fitted with a skewed normal distribution. Insert: pairwise distribution plot of the difference between the two end-to-end distance populations. Histogram was fitted with a Gaussian distribution.

To determine the effect of specific DNA sequences, we carried out TPM experiments with a 685 bp DNA substrate with the Clone20 sequence at its center. The reduction of the root mean square displacement (RMS) of the DNA tether in TPM indicates that both HMfA and HMfB compact the Clone20 substrate (Figures 1A,B). Compaction as a function of protein concentration occurs in two steps. The compaction step at high protein concentrations (at > ~30 nM for both HMfA and HMfB) resembles the strong cooperative compaction of nonspecific DNA into a hypernucleosome (Figures 1A,B). This step occurred at slightly higher protein concentrations on Clone20 DNA than on nonspecific DNA. The first compaction step, occurring at low protein concentrations (at 1–30 nM for HMfA and 20–30 nM for HMfB), was not observed for nonspecific DNA, and is therefore due to specific binding of HMfA and HMfB to the Clone20 sequence. At this step, the RMS is reduced to ~125 nm. For HMfB, this state is unpopulated up to 20 nM, partially populated at 20–22 nM and completely populated at 23–25 nM. The ratio of both populations is expressed as occupancy for HMfB, to which the Hill equation was fit (Figure 1C). This resulted in a binding constant of (K_D_) of 21 ± 0.2 nM and a Hill binding coefficient (*n*) of 32 ± 8. HMfA directly fully populates this intermediate state at 1–30 nM (Figure 1A). Therefore, no exact binding constant could be calculated as the intermediate state is already fully populated at 1 nM, which means that the binding constant of HMfA for Clone20 is in the sub-nanomolar concentration range. The Clone20 site consists of 60 bp, theoretically permitting binding of a tetramer to this sequence. To determine whether this is indeed the case, we calculated the end-to-end distance of the DNA molecule without protein and with 5 nM HMfA (Figure 1D). This resulted in an end-to-end distance of 101 ± 11 nm and 78.9 ± 11 nm for 0 and 5 nM, respectively. The pairwise distribution gives a difference of 22.8 ± 10 nm, corresponding to 67 ± 30 bp (where each bp is 0.34 nm). The same analysis was done for HMfB at a concentration of 21 nM, where two populations were observed (Supplementary Figure S1), and this yielded a difference of 23.0 ± 9 nm or 68 ± 27 bp. These observations suggest that both HMfA and HMfB form a structurally identical tetrameric histone-DNA complex at the Clone20 site. However, this site is unable to act as a nucleation site as it does not promote hypernucleosome formation.

The finding that HMfA exhibits a higher binding affinity for Clone20 than HMfB contradicts results from EMSA experiments (Bailey et al., 2002). The difference may be caused by a different pH [7.0 in our experiments vs. 8.0 in the studies of Bailey et al., (2002)] as the isoelectric points of HMfA and HMfB are different (8.06 and 9.59, respectively). Another possibility is that a difference in measured affinity is a result of using different methods, with EMSA involving a gel matrix and TPM using DNA in solution attached to a glass surface. Also, the DNA substrate length is different; our 685 bp substrate is much longer than the 110 bp used by Bailey et al., which could have effects on apparent binding affinity and cooperativity.

### 3.2. The Clone20 DNA sequence is recognized by HMfA/B derivatives impaired in hypernucleosome formation

**Figure 2:**
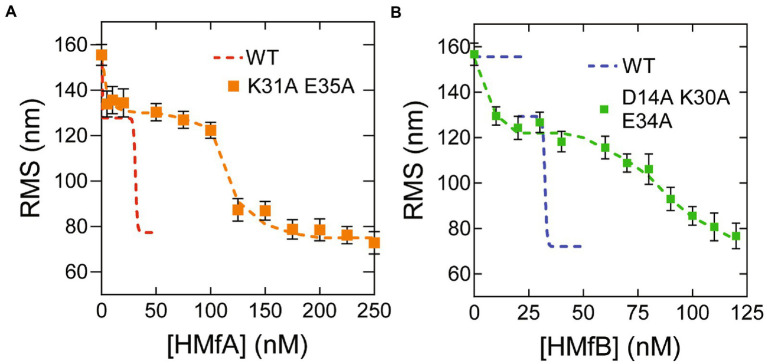
Histone derivatives HMfAK31A E35A and HMfBD14A K30A E34A recognize the Clone20 sequence. **(A)** Root mean square displacement (RMS) values of Clone20 DNA tethers with HMfA_K31A E35A_ or **(B)** HMfB_D14A K30A E34A_ as measured by TPM in 50 mM Tris–HCl pH 7, 75 mM KCl. Wildtype data was reproduced from Figure 1. Histograms were fitted to a Gaussian distribution. Error bars represent the propagated standard deviation of at least two replicates. Dashed lines are to guide the eye.

Previously, we found that the HMfA and HMfB derivatives HMfA_K31A E35A_ and HMfB_D14A K30A E34A_ require higher concentrations to fully compact nonspecific DNA and that the resulting hypernucleosome is less stable compared to the wildtype, especially for HMfB (Henneman et al., 2021). These observations underscore the importance of the mutated residues in stabilizing hypernucleosome structure via electrostatic interactions between hypernucleosomal stacks. These HMfA and HMfB derivatives have additional relevance as mimics of histone variants from other species that lack stacking interactions (Henneman et al., 2018b). We examined if these proteins still exhibit specific binding to the Clone20 sequence. Both derivatives compact the Clone20 DNA into a tetramer at comparable protein concentrations as the wildtype proteins (Figures 2A,B). This result indicates that HMfA and HMfB recognize the Clone20 site independent of their stacking interactions, as expected. Nonspecific binding, leading to hypernucleosome formation occurs at >125 nM for HMfA_K31A E35A_ and > 80 nM for HMfB_D14A K30A E34A_. These concentrations are higher than observed from the wildtype proteins, which indicates delayed hypernucleosome formation attributed to the missing stacking interactions. Also the transition from tetramer to hypernucleosome is more gradual for both histone derivatives than for the wildtype proteins. The distinct binding at a specific DNA sequence by archaeal histones at concentrations below the effective K_D_ for nonspecific compaction implies that specific sites may have a functional role in archaea. Also, the difference in affinity for the Clone20 sequence between HMfA and HMfB (and their mutated derivatives) supports the hypothesis that histone variants have distinct functional roles, potentially in transcription regulation (Sandman and Reeve, 2006; Henneman et al., 2018b; Stevens et al., 2020).

### 3.3. Histone tetramers have increased affinity for Clone20 and can bind in different conformations

**Figure 3:**
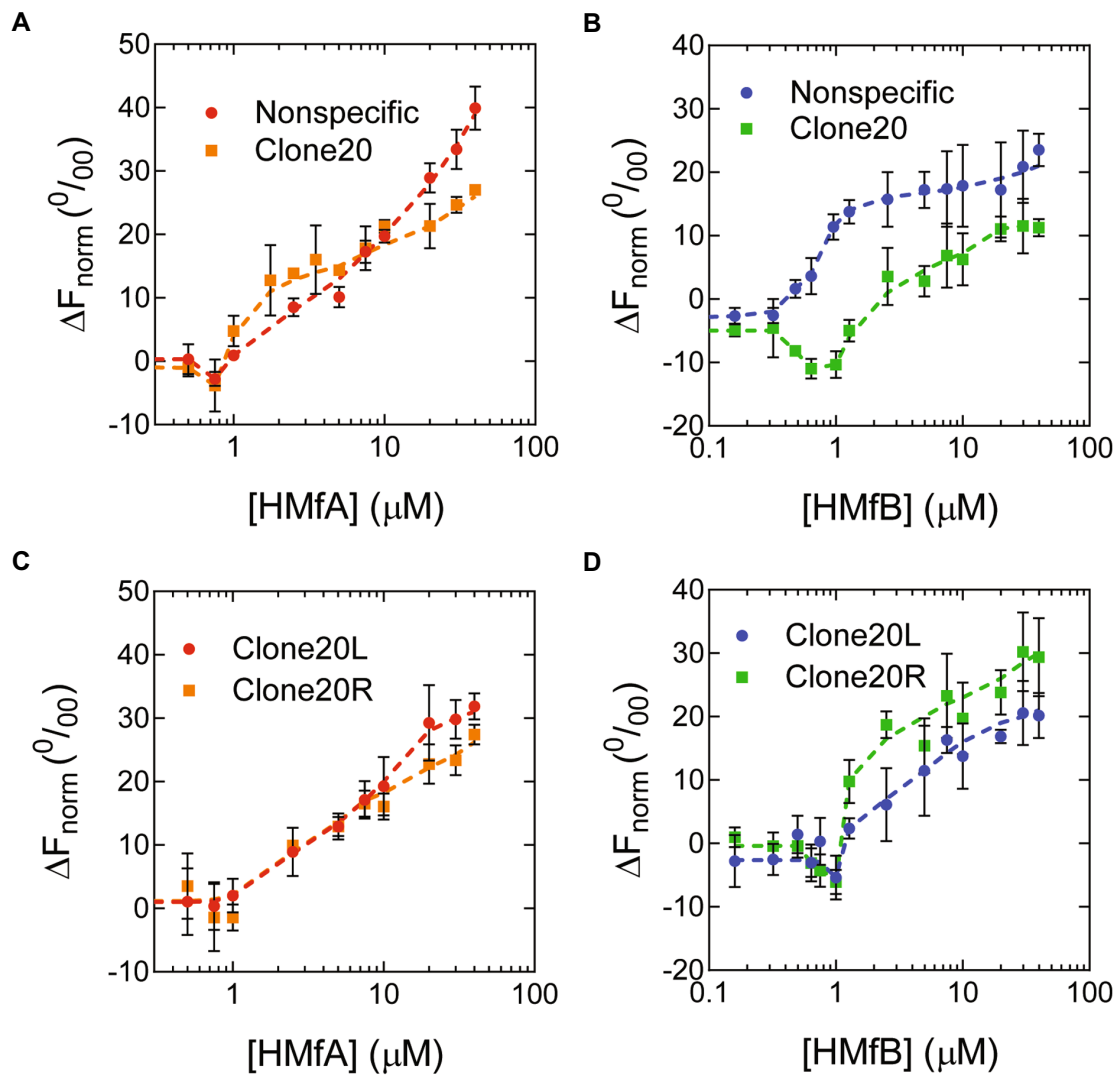
Binding of HMf proteins to short DNA substrates using Microscale Thermophoresis. Normalized thermophoresis curves of Nonspecific or Clone20 DNA as a function of **(A)** HMfA or **(B)** HMfB or of Clone20L or Clone20R as a function of **(C)** HMfA or **(D)** HMfB. Error bars indicate the standard deviation of three independent measurements. Dashed lines are lines to guide the eye.

To further investigate the properties and affinities of the respective tetramers formed on the different DNA sequences, we used microscale thermophoresis (MST) with short (78 bp) DNA substrates designed to accommodate maximally two HMf dimers (Figure 3; Supplementary Figure S2) and fitted the binding curves with the Hill binding model (Supplementary Figure S3). For HMfA, the affinity for Clone20 DNA is higher than for nonspecific DNA, while the cooperativity stayed the same (Table 1). Judged by the in general higher ΔF_norm_ for HMfA compared to HMfB, the protein-DNA complexes formed by HMfB are more compact than those formed by HMfA (Figures 3A,B). This agrees with earlier observations where the hypernucleosome formed by HMfB is more compact and has a higher stacking energy than that formed by HMfA (Henneman et al., 2021). Also the ΔF_norm_ at the highest protein concentration of nonspecific DNA is higher than that of Clone20 for both proteins, indicating a more compact structure formed on the specific site.

**Table 1:** Binding affinities (K_D_) and Hill binding coefficients (h) of HMf to 78 bp DNA substrates. The values were determined by fitting of the MST data to the Hill binding model.

Protein	DNA substrate	K_D_ (μM)	h
HMfA	Nonspecific	16.5 ± 6.7	1.08 ± 0.19
	Clone20	3.75 ± 0.10	1.08 ± 0.21
	Clone20L	8.09 ± 1.4	1.23 ± 0.16
	Clone20R	7.34 ± 2.8	0.992 ± 0.21
HMfB	Nonspecific	0.915 ± 0.089	3.30 ± 0.97
	Nonspecific +0.2% Tween20	19.6 ± 5.4	1.22 ± 0.15
	Clone20 specific	0.243 ± 0.16	1.24 ± 0.91
	Clone20 nonspecific	2.99 ± 0.81	1.44 ± 0.47
	Clone20L specific	n.a.	n.a.
	Clone20L nonspecific	4.11 ± 1.0	1.30 ± 0.38
	Clone20R specific	0.660 ± 0.047	7.45 ± 3.5
	Clone20R nonspecific	12.3 ± 27	0.564 ± 0.30
HMfA _K31A E35A_	Nonspecific	n.a.	n.a.
	Clone20	n.a.	n.a
HMfB _D14A K30A E34A_	Nonspecific +0.2% Tween 20	22.0 ± 0.71	4.09 ± 0.62
	Clone20 + 0.2% Tween 20	19.6 ± 1.194	3.51 ± 0.77

HMfB exhibited two-step behavior on Clone20 DNA (Figure 3B). The first state, attributed to specific binding to the Clone20 site, resulted in a negative ΔF_norm_, so a more compact structure compared to unbound DNA. While a slight decrease in ΔFnorm was observed for HMfA as well (Figure 3A), it was less pronounced than for HMfB and we were unable to fit any binding constant. The second state showed increasing ΔF_norm_ and corresponds to nonspecific binding. There are multiple possibilities to explain this two-step behavior. An HMfB tetramer could bind first, forming a compact bent structure. At higher protein concentrations, a hexamer with suboptimal protein-DNA interaction interface might assemble on the DNA. This would be a metastable structure as the DNA substrate is shorter than expected for hexamer binding (78 bp compared to 90 bp theoretically). Another option might be binding of an HMfB dimer, which bends the DNA resulting in the observed compact structure. The second binding regime would then represent tetramer (or even hexamer) formation on the DNA substrate.

In order to be able to distinguish between the two possible models described above, we performed MST experiments with derivatives of the Clone20 DNA substrate, where only either the left (Clone20L) or the right (Clone20R) site of the sequence is present (Supplementary Table S1). The other half was replaced with the nonspecific DNA sequence. For HMfA, this leads to a generally lower affinity than for the entire Clone20 sequence but higher than for nonspecific DNA (Figure 3C; Table 1). HMfB still shows the two-step binding behavior mainly on Clone20R (Figure 3D; Table 1). This suggests that either a dimer is binding and therefore half of the Clone20 sequence is sufficient, or that half the site is enough to position a tetramer on the DNA. Strikingly, the ΔFnorm at the highest HMfB concentration increased compared to the fully nonspecific and Clone20 substrates, especially for Clone20R. This means that the resulting structure is less compact or the DNA is more permissive to HMfB multimerization. TPM experiments with only Clone20L or Clone20R present were in agreement with the MST experiments (Supplementary Figure S4). For HMfA, tetramer binding cannot be observed for both half sites; instead, HMfA shows similar binding behavior as on nonspecific DNA (Supplementary Figure S4A). Tetrameric complex formation by HMfB, as observed by having two populations (Figure 1B), was only found on Clone20R (Supplementary Figure S4B), but with a slightly reduced affinity compared to the full Clone20 site (Kd of 28.8 ± 1.1 nM versus 21 ± 0.2 nM; Supplementary Figure S4C). We calculated the end-to-end distance of the two observed populations and found a pairwise distance of 27.6 ± 11 nm or 81 ± 32 bp, confirming that a tetramer is most likely bound to the Clone20R site (Supplementary Figure S4D). The RMS of Clone20L for 10–30 nM HMfB is slightly lower than unbound Clone20R DNA, but higher than the second population corresponding to the tetrameric complex (Supplementary Figure S4B). This could be suggestive of binding of a dimer, but the resolution of TPM experiments is not high enough to confirm this.

MST experiments with the HMf derivatives showed that HMfA_K31A E35A_ had too low an affinity for both DNA substrates to be reliably fitted (Supplementary Figures S2A, S3). HMfB_D14A K30A E34A_ showed increased aggregation in MST experiments; therefore, 0.2% Tween20 had to be added (Supplementary Figure S2B). Most likely, this is an artifact of using protein concentrations in the micromolar range for MST experiments in comparison to nanomolar for TPM. To be able to compare, also an HMfB wildtype titration with nonspecific DNA was done in the presence of 0.2% Tween20. The affinities of HMfB_D14A K30A E34A_ for both DNA substrates are similar (Table 1) and qualitatively the curves are also comparable. No two-step behavior was observed on the Clone20 DNA substrate.

## 4. Discussion

**Figure 4:**
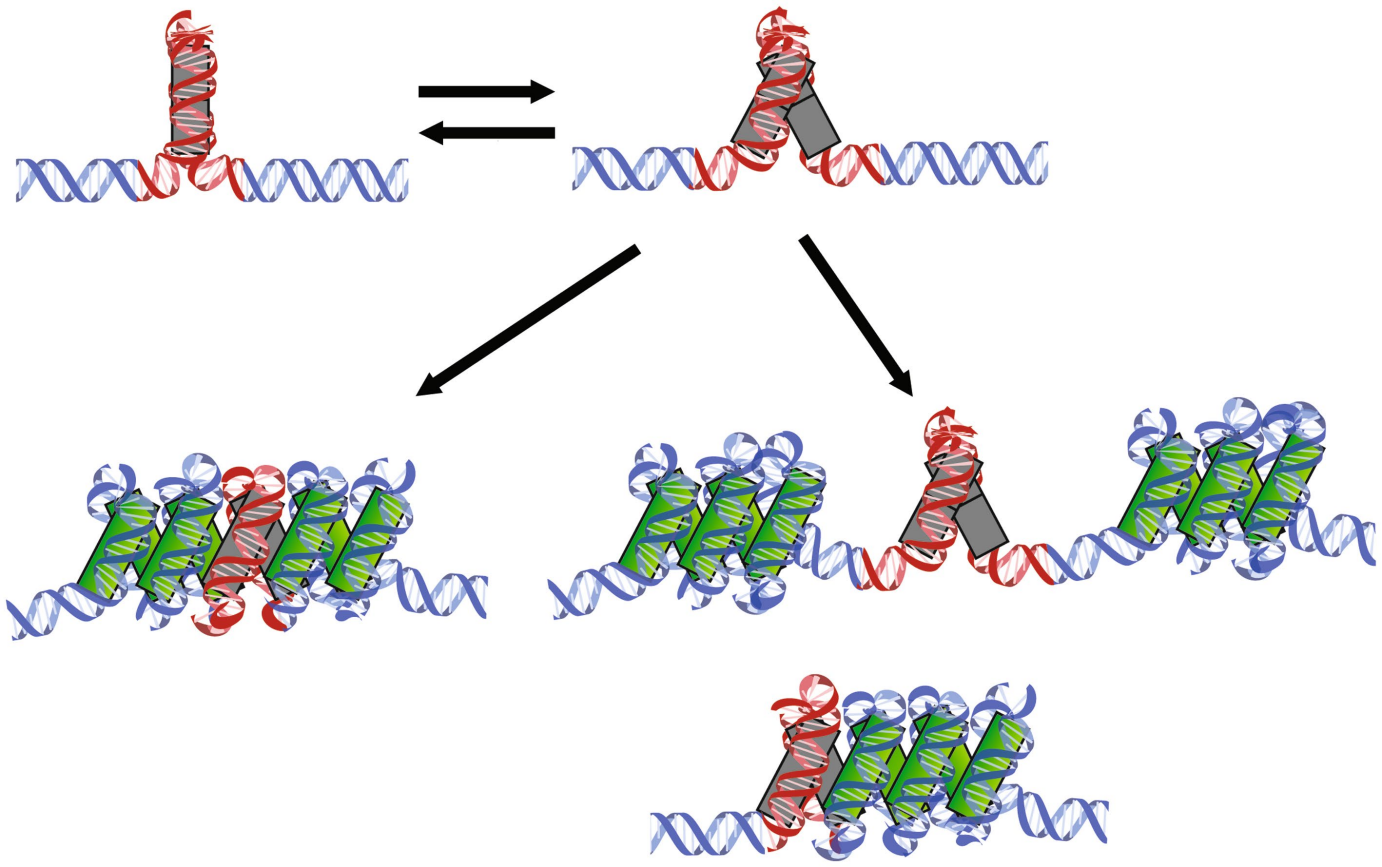
Mechanisms of HMf tetramers binding to specific DNA sequences followed by hypernucleosome formation. HMf tetramers bind to the Clone20 sequence and form a closed complex incompatible with further multimerization (top left). This structure can dynamically open and close (top right). The open structure can facilitate hypernucleosome formation (bottom left). If histone variants are bound that lack either stacking interactions or dimer-dimer interactions are bound, this tetramer could potentially act as a barrier of hypernucleosome progression or act as a ‘capstone’ (bottom right). Including different homo-and heterodimers into one structure could also result in limited extension of the hypernucleosome.

A DNA substrate containing the artificial high-affinity sequence Clone20 is compacted by *M. fervidus* histones in two distinct steps, representing two distinct types of complexes. HMf is a dimer in solution, even at high concentrations above 1 mg/ml (Supplementary Figure S5). We propose a model where the first step is binding of a dimer to the DNA, directly followed by recruitment of the second dimer to form a stable tetrameric complex. Recruitment of the second dimer is cooperative due to interactions with both DNA and the dimer already bound to the DNA. We found that the tetramer on the Clone20 site exists in a distinct structural, possibly more closed, state incompatible with hypernucleosome formation. Therefore the high affinity sequence is unable to act as a nucleation site. This closed state is in equilibrium with the more open state, which is geometrically permissive for multimerization (Figure 4). On nonspecific DNA, most likely only open tetramers can bind, which explains why such dynamics at the dimer-dimer interface were not observed with molecular dynamics simulations of HMfB (Bowerman et al., 2021).

Globally, archaeal histone variants can be divided into three functional groups. The first group consists of histones that contain the amino acid residues involved in both dimer-dimer interactions (tetramer formation) and stacking interactions (hypernucleosome formation). Members of this group include the archaeal model histones HMfA and HMfB, and HTkB from *Thermococcus kodakarensis* (Henneman et al., 2018b). Generally, they show cooperative extension on DNA, resulting in hypernucleosome formation once the first tetramer is in the right position (Figure 4). However, differences in DNA binding properties between members of this group do exist, and environmental or growth phase related response may bias the expression of one histone variant over another, resulting in changes in (local) chromosome organization, potentially translating into an altered expression of genes (Sandman et al., 1994). Hypernucleosome formation by HMfB is more cooperative than for HMfA, and also the level of DNA compaction achieved is slightly higher (Henneman et al., 2021). HMfA, on the other hand, has a higher affinity for the Clone20 sequence in the context of longer DNA (Figure 1C). This finding was unexpected as the Clone20 sequence was obtained *via* SELEX optimization with HMfB. Nevertheless, this finding may be indicative of distinct functions in chromosome organization. HMfA may more effectively position tetramers at specific locations on the genome, setting boundaries for hypernucleosome formation and the action of other chromatin proteins, whereas HMfB forms predominantly hypernucleosomes. However, this is contradicted by experiments on shorter DNA, such as in Bailey et al. (2002) and in our MST experiments (Figure 3). Bailey et al. found that the difference in affinity between HMfA and HMfB was at least partially dependent on the C-terminal residues of helix α3, which does not make direct contact with the DNA, but is important in dimer-dimer interactions (Decanniere et al., 2000). Also, it has been proposed before that changes in the dimer-dimer interface might result in tetramers that bend the DNA with either a negative or positive supercoil akin to the eukaryotic (H3-H4)2 tetramer (Hamiche et al., 1996; Hamiche and Richard-Foy, 1998; Sandman and Reeve, 2000). Potentially, this interface is involved in forming the closed and open conformation of the HMf tetramer, proposed here (Figure 4). This would require extensive structural follow-up studies on the different protein-DNA complexes. The genomic context and amount of other proteins bound to the DNA might be of importance. Synergistic or antagonistic interplay between histones and other architectural proteins could be expected, but has not been studied in detail so far.

The second group of histone variants consists of histones that are able to form dimers and tetramers, but lack the stacking interactions implied in the stabilization of hypernucleosomes. Examples are the histone derivatives HMfA_K31A E35A_ and HMfB_D14A K30A E34A_ and the *Haloredivivus* sp. G17 and *Methanococcoides methylutens* histones (Henneman et al., 2018b). They are able to recognize a specific DNA sequence in a similar concentration range as histones from the first group (Figure 2), but hypernucleosome formation will occur at higher concentration and less cooperatively due to the absence of stabilizing stacking interactions. The presence of a tetramer formed by these histones could act as a roadblock for hypernucleosome progression or act as a capstone by preventing further multimerization on one side of the hypernucleosome (Figure 4). In this way, changing expression levels of histone variants might affect DNA compaction and potentially transcriptional regulation.

The last group of histone variants lacks the residues implied in dimer-dimer interactions. Therefore, these histones are likely bound as dimers only or, when incorporated in a heterodimer, prevent a hypernucleosome from further multimerization and thus acting as capstones (Stevens et al., 2020). They may have intact stacking interactions, potentially permitting the formation of hypernucleosomes (of reduced stability compared to the model histones HMfA and HMfB). Some predicted members of this group are *Ca. Lokiarchaeota* GC14_75 HLkE and *Nanosalina* J07AB43 HB (13).

Clone20 can be regarded as the archaeal counterpart of the 601 nucleosome positioning sequence, a sequence that energetically favors nucleosome formation. The 601 sequence is often used in studies on eukaryotic nucleosomes (Lowary and Widom, 1998; Thåström et al., 1999; Vasudevan et al., 2010; Eslami-Mossallam et al., 2016). However, sequences with high similarity to Clone20 and 601 sequences have thus far not been identified in genomes, and affinity for the 601 sequence was found to be much higher than for natural sequences (van der Heijden et al., 2012). Based on our results of HMfB binding to the right site of Clone20 (Figure 3D; Supplementary Figure S4), it might be possible that a smaller site is sufficient to act as a high-affinity sequence. This would increase the possibility of encountering such a sequence in genomes.

Taken together, the interplay between archaeal histone variants and specific genomic sequences can result in the formation of structurally different protein-DNA complexes. Positioning of these complexes along the genome might have a potential to act in archaeal transcription regulation.

## Data availability statement

The datasets generated during the current study are available from the 4TU repository (https://data.4tu.nl) with https://doi.org/10.4121/22047197.v1.

## Author contributions

AE, BH, RV, and NK performed the experiments. AE, BH, RV, NK, and RD contributed to data analysis and discussion. AE, BH, RV, and RD wrote, reviewed, and corrected the manuscript. All authors contributed to the article and approved the submitted version.

## Funding

This work was supported by the Netherlands Organization for Scientific Research [VICI 016.160.613/533 and OCENW.GROOT.2019.012 to RD].

## Conflict of interest

The authors declare that the research was conducted in the absence of any commercial or financial relationships that could be construed as a potential conflict of interest.

## Publisher’s note

All claims expressed in this article are solely those of the authors and do not necessarily represent those of their affiliated organizations, or those of the publisher, the editors and the reviewers. Any product that may be evaluated in this article, or claim that may be made by its manufacturer, is not guaranteed or endorsed by the publisher.
