# Supplementary material for: Specific DNA binding of archaeal histones HMfA and HMfB
Source: Front Microbiol. 2023 Apr 18;14:1166608. doi: 10.3389/fmicb.2023.1166608 (PMC10151503; doi:10.3389/fmicb.2023.1166608)
Supplement: Supplementary file 1 [file Data_Sheet_1.PDF]

Supplementary Material

Specific DNA binding of archaeal histones HMfA and HMfB

Amanda M. Erkelens[1†], Bram Henneman[1†], Ramon A. van der Valk[1], Nancy C.S. Kirolos[1] & Remus T. Dame[1,2,*]

*** Correspondence:** R.T. Dame rtdame@chemleidenuniv.nl

Table S1: Sequences of DNA substrates used for MST experiments. The Cy5-label is attached on the '5-end of the top strand. For Clone20L and Clone20R, the nonspecific part is indicated in black and part of the Clone20 substrate in red.

Name DNA substrate	Sequence (5'-3')
Nonspecific	CGGCGCAAATTCGTGACCAGTTGCATCAGCTGCGTGAGCTGTTTAT CGCAGCATCGTAACAGGATAGTGAAGAAGACT
Clone20	GGATCCCTGTCGGCACAGTTGAGCGATCAAAAACGCCGTAGAACG CTTTAATTGATAATCAAAGGCCGCAGAGAGCTC
Clone20L	GGATCCCTGTCGGCACAGTTGAGCGATCAAAAACGCCGTAGATTA TCGCAGCATCGTAACAGGATAGTGAAGAAGACT
Clone20R	CGGCGCAAATTCGTGACCAGTTGCATCAGCTGCGTGCGTAGAACG CTTTAATTGATAATCAAAGGCCGCAGAGAGCTC

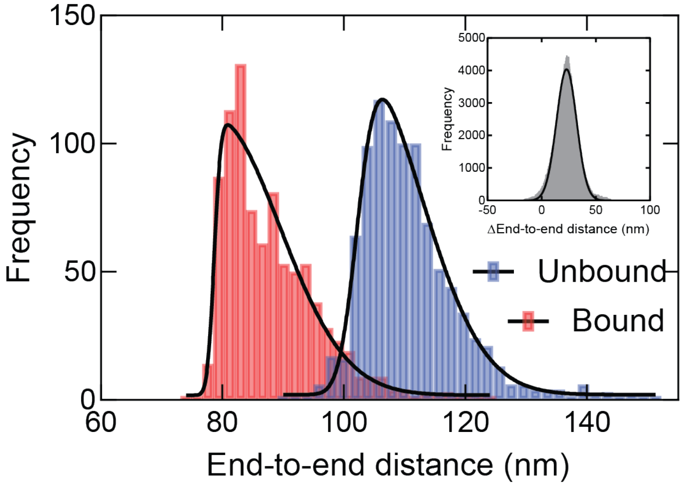

Figure S1 Calculated end-to-end distances for the unbound and bound population of 21 nM HMfB on Clone20 DNA. Histograms were fitted with a skewed normal distribution, resulting in end-to-end distances of 102 ± 10 nm and 78.6 ± 11 nm for unbound and bound DNA respectively. Insert: pairwise distribution plot of the differences between the two end-to-end distance peaks. Histogram was fitted with a Gaussian distribution resulting in a difference of 23.0 ± 9.3 nm.

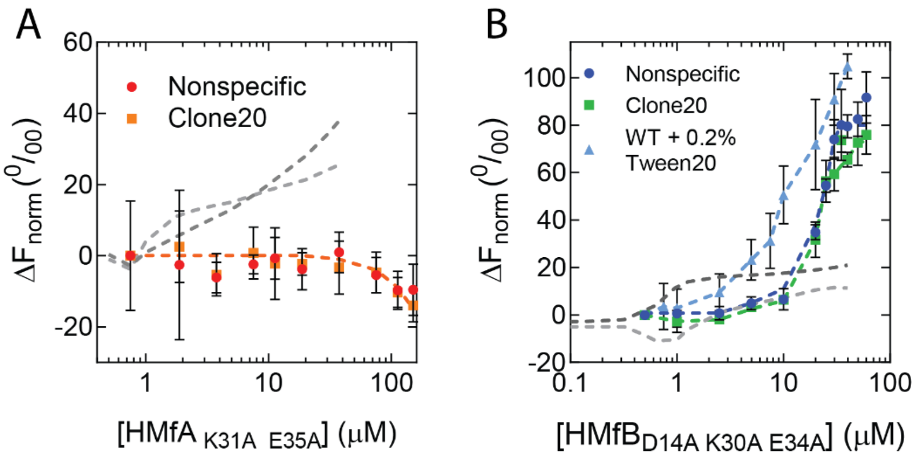

Figure S2: Binding of HMf derivatives to Nonspecific and Clone20 DNA substrates. Normalized thermophoresis curves of Nonspecifc or Clone20 DNA as a function of A) $HMfA_{K31A\ E35A}$ or B) $HMfB_{D14A\ K30A\ E34A}$. For HMfB WT, an extra curve on Nonspecific DNA was measured with 0.2% Tween20 in the buffer. Error bars indicate the standard deviation of three independent measurements. Dashed lines are lines to guide the eye. Wildtype curves from WT proteins (figure 3) are included in dark (Nonspecific) and light (Clone20) grey for easy comparison.

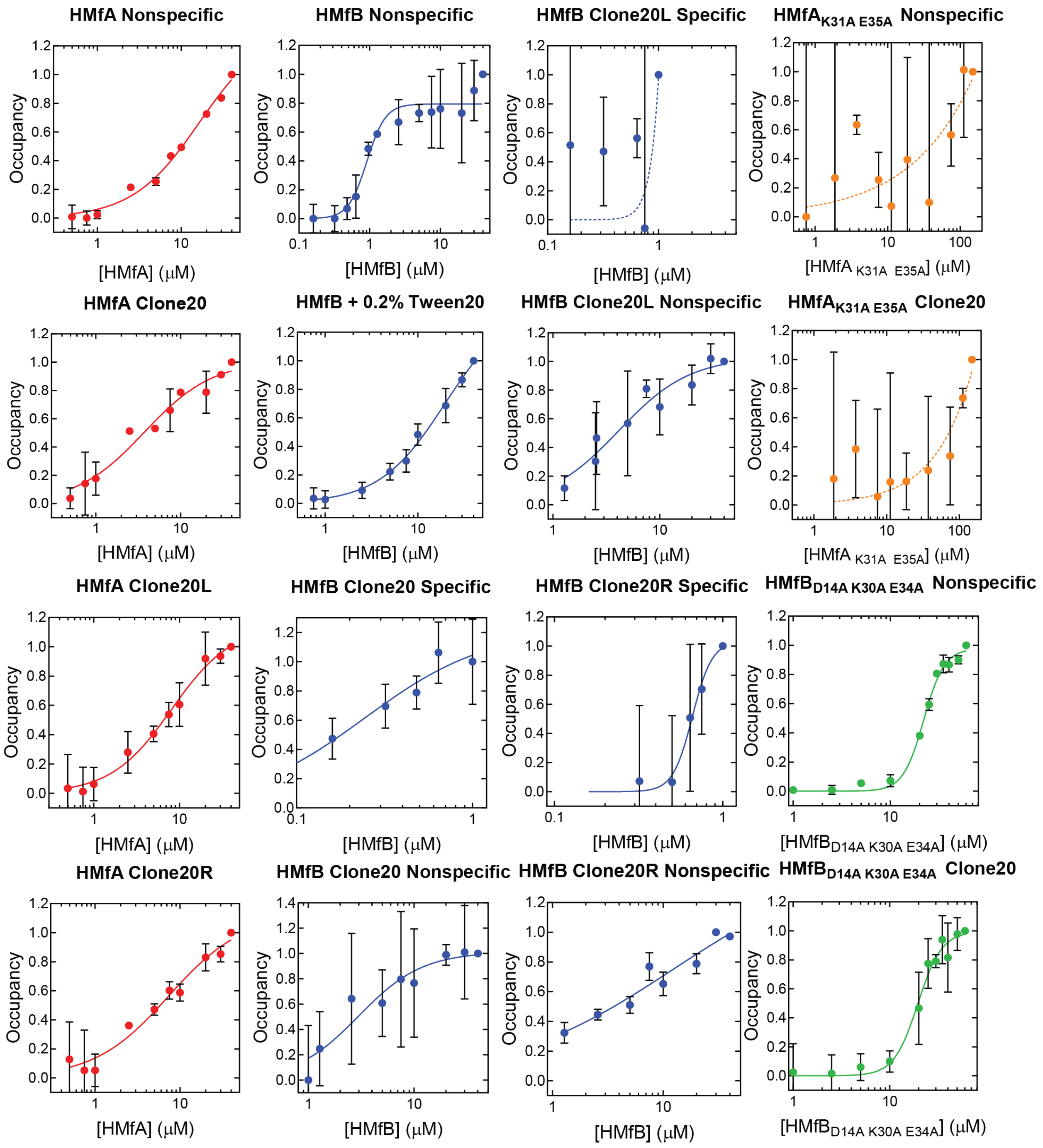

Figure S3: Hill fits of MST curves Occupancy was fitted against protein concentration where the occupancy at the highest concentration was set as 1.0. The dotted lines indicate fits that did not result in any reliable results.

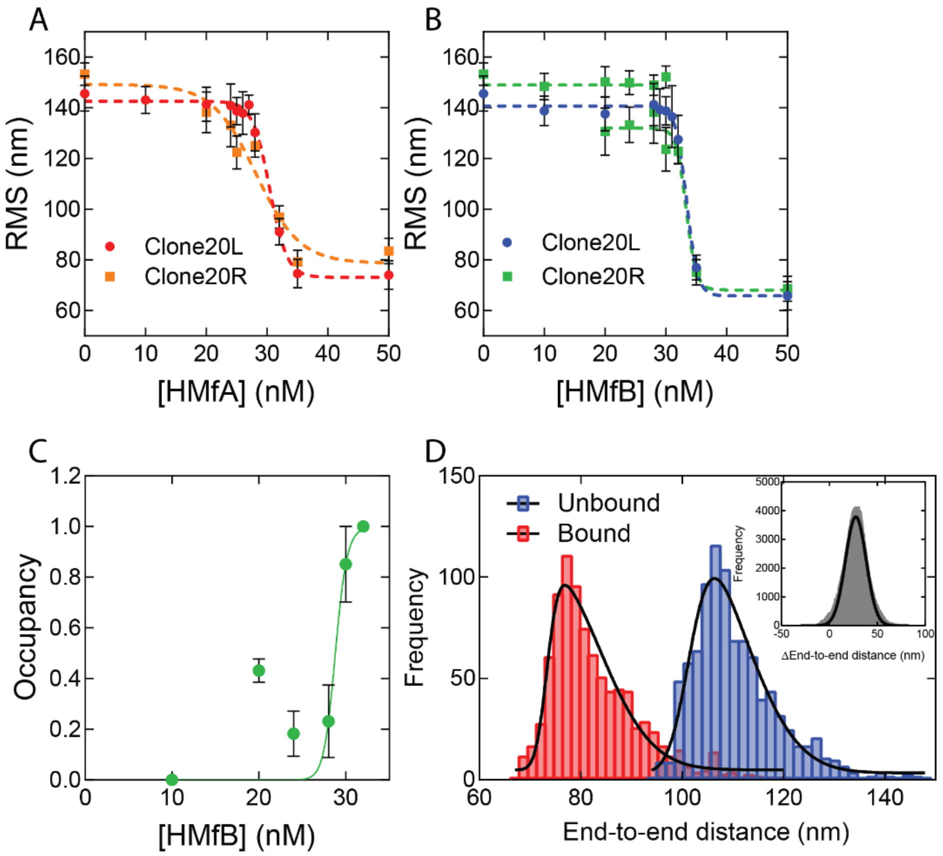

Figure S4 Binding of HMfA and HMfB to Clone20L or Clone20R in TPM experiments. Root mean square displacement (RMS) of Clone20L and Clone20R DNA incubated with A) HMfA or B) HMfB in 50 mM Tris-HCl pH 7, 75 mM KCl. Histograms were fitted to a Gaussian distribution. Error bars represent the propagated standard deviation of two replicates. Dashed lines are lines to guide the eye. C) Binding curve of HMfB on Clone20R DNA. Data point were fitted using the Hill binding model. D) Calculated end-to-end distance for bound and unbound Clone20R DNA incubated with 30 nM HMfB. Histograms were fitted with a skewed normal distribution. Insert: pairwise distribution plot of the difference between the two populations. Histogram was fitted with a Gaussian distribution.

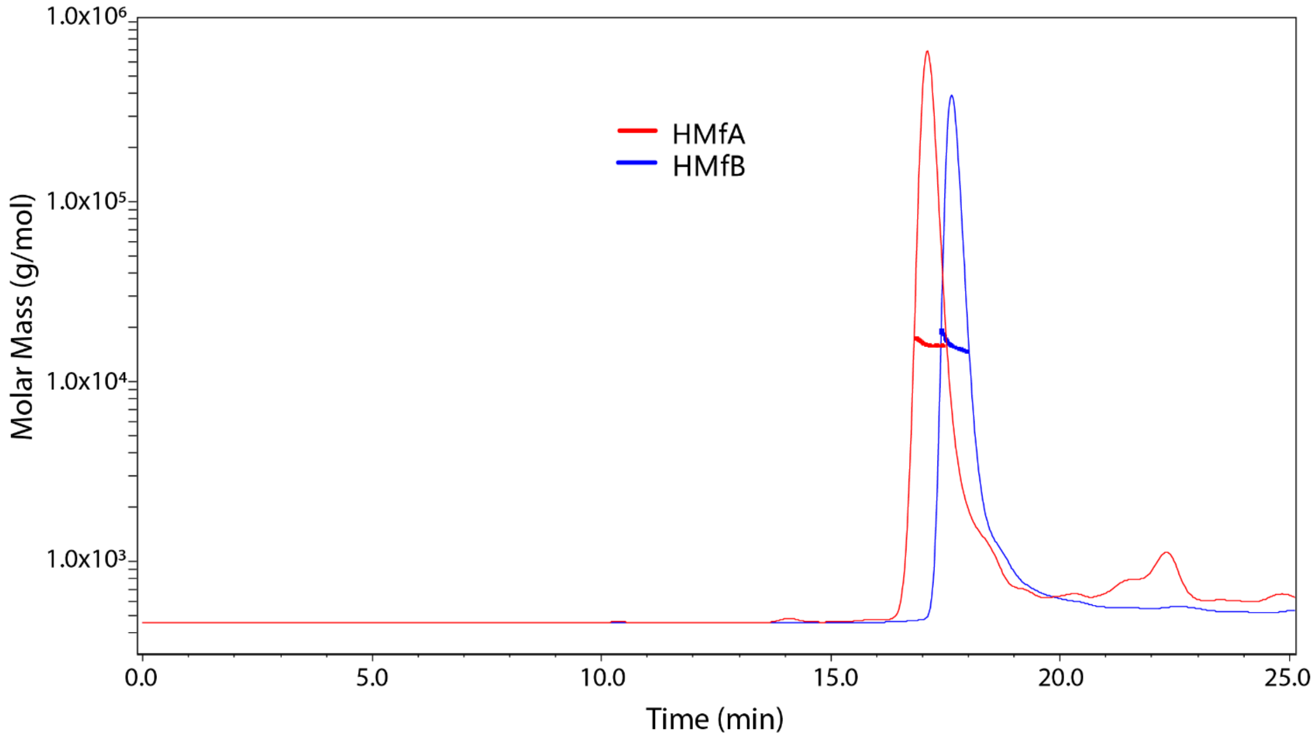

Figure S5 HMfA and HMfB are dimers in solution. SEC-MALS result of at least 1 mg/ml HMfA and HMfB. The determined molecular weight for HMfA was 16.3 ± 0.3 kDa and for HMfB 16.0 ± 0.1 kDa. The theoretical monomer mass of HMfA is 7.5 kDa and for HMfB 7.7 kDa.
